# National Outcomes of Elective Hybrid Arch Debranching with Endograft Exclusion versus Total Arch Replacement Procedures: Analysis of the Society of Thoracic Surgeons Adult Cardiac Surgery Database

**DOI:** 10.1055/s-0041-1724003

**Published:** 2021-10-04

**Authors:** Tyler Wallen, Timothy Carter, Andreas Habertheuer, Vinay Badhwar, Jeffrey P. Jacobs, Babatunde Yerokun, Amelia Wallace, Karianna Milewski, Wilson Y. Szeto, Joseph E. Bavaria, Prashanth Vallabhajosyula

**Affiliations:** 1 Division of Cardiovascular Surgery, The University of Florida, Gainesville, Florida; 2 Division of Cardiovascular Surgery, The University of Pennsylvania Health System, Philadelphia, Pennsylvania; 3 Division of Cardiac Surgery, West Virginia University Heart and Vascular Institute, Morgantown, West Virginia; 4 Duke University Medical Center, Durham, North Carolina; 5 Division of Cardiothoracic Surgery, Yale University School of Medicine, New Haven, Connecticut

**Keywords:** thoracic, aorta, hybrid, aneurysm

## Abstract

**Objective** Hybrid arch procedures (arch vessel debranching with thoracic endovascular aneurysm repair [TEVAR] coverage of arch pathology) have been presented as an alternative to total arch replacement (TAR). But multicenter-based analyses of these two procedures are needed to benchmark the field and establish areas of improvement.

**Methods** The Society of Thoracic Surgeons (STS) Adult Cardiac Surgery Database from July 2014 to December 2015 was queried for elective TAR and hybrid arch procedures. Demographics and operative characteristics were compared and stepwise variable selection was used to create a risk-set used for adjustment of all multivariable models.

**Results** A total of 1,011 patients met inclusion criteria, 884 underwent TAR, and 127 had hybrid arch procedures. TAR patients were younger (mean age: 62.7 ± 13.3 vs. 66.7 ± 11.9 years; *p* = 0.001) and had less peripheral vascular disease (34.0 vs. 49.6%; *p* < 0.001) and preoperative dialysis (1.7 vs. 4.7%; *p* = 0.026), but similar history of stroke ( *p* = 0.91)/cerebrovascular disease ( *p* = 0.52). TAR patients had more concomitant procedures (60 vs. 34.6%; *p* < 0.0001). TAR patients had lower mortality (6.7 vs. 12.6%; *p* = 0.02), stroke (6.9 vs. 15%; *p* = 0.002), paralysis (1.8 vs. 7.1%; *p* = 0.002), renal failure (4.6 vs. 8.7%; *p* = 0.045), and STS morbidity (34.2 vs. 42.5%; *p* = 0.067). Composite mortality, stroke, and paralysis were significantly lower with TAR (11.5 vs. 25.2%; *p* = 0.0001). After risk adjustment, analysis showed hybrid arch procedures imparted an increased odds of mortality (odds ratio [OR] = 1.91, *p* = 0.046), stroke (OR = 2.3, *p* = 0.005), and composite endpoint of stroke or mortality (OR = 2.31, *p* = 0.0002).

**Conclusion** TAR remains the gold standard for elective aortic arch pathologies. Despite risk adjustment, hybrid arch procedures were associated with increased risk of mortality and stroke, advocating for careful adoption of these strategies.

## Introduction

The gold standard for the management of aortic arch aneurysm has been surgical total arch replacement (TAR) and the frozen elephant trunk techniques. 1 2 3 The advent of endovascular stent graft technology for descending thoracic aortic (DTA) pathologies has led to extrapolation of its principles to the aortic arch. This application, termed “hybrid arch” or “arch hybrid” procedure, has been reported to be performed under elective and urgent/emergent conditions in a wide array of nuanced and self-tailored techniques. Furthermore, translation of thoracic endovascular aneurysm repair (TEVAR) technology to the aortic arch has led to expansion of the indications for performing more extensive operations, without any evidence from randomized trials comparing traditional open to hybrid arch procedures, although several centers have reported good outcomes in retrospective analyses. For example, the availability of a hybrid total arch graft with built-in endograft extension has been applied for empiric treatment of DeBakey Type-I aortic dissection to achieve long-term stabilization and improved remodeling of the DTA, at times even when the entry tear is not in the aortic arch. 4 In this context, as a large volume aortic center embracing TEVAR technology, our own institution has reported outcomes with transverse hemiarch reconstruction with antegrade TEVAR of the DTA for DeBakey Type-I aortic dissection, even though no randomized trial has shown the benefit of antegrade TEVAR in this clinical setting. 5

As the surgical options for management of aortic arch pathologies continue to evolve and diversify, the Society of Thoracic Surgeons (STS) Adult Cardiac Surgery Database was queried to investigate outcomes associated with adoption of hybrid arch procedures. Outcomes were then compared with TAR procedure as the gold standard to benchmark the field and establish areas for improvement.

## Materials and Methods

### Patient Selection

**Fig. 1:**
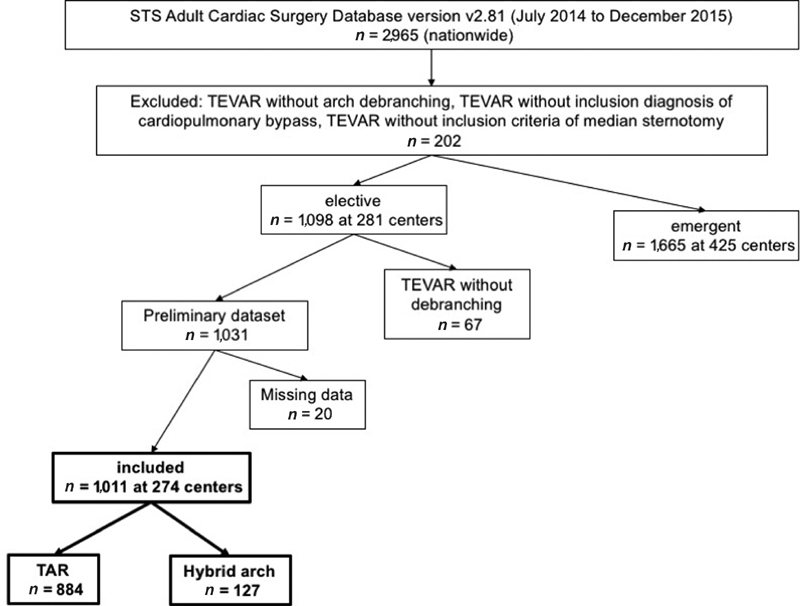
Study population. STS, the Society of Thoracic Surgeons; TAR, total arch replacement; TEAVAR, thoracic endovascular aneurysm repair.

**Fig. 2:**
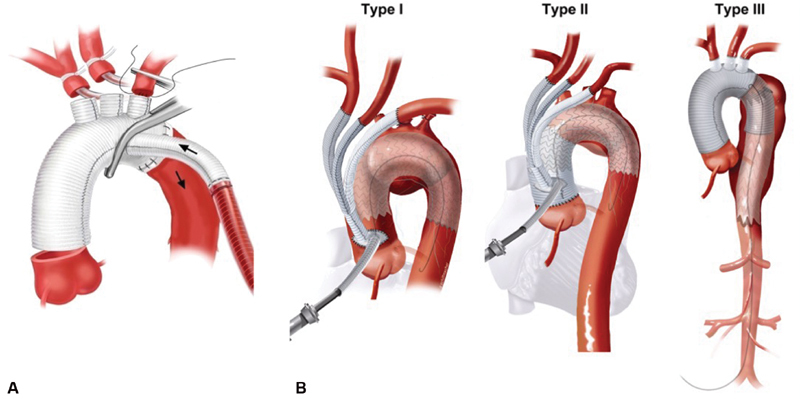
Technical aspects of surgical reconstruction for total aortic arch replacement and hybrid arch procedures: panel ( **A** ) represents traditional total aortic arch replacement with a trifurcated graft which requires the use of deep hypothermic circulatory arrest; panel ( **B** ) represents various configurations of hybrid aortic arch replacement. Type I includes cerebrovascular debranching followed by aortic endografting through a side arm. Type II includes ascending aorta replacement, cerebrovascular debranching, and aortic endografting through a side arm. Type III includes ascending aorta replacement, aortic arch replacement, and antegrade (under circulatory arrest), or retrograde endografting.

The STS Adult Cardiac Surgery Database version v2.81 from July 2014 to December 2015 was queried for TAR/repair cases as this version of the database and timeframe captured all preoperative inclusion criteria accurately. This resulted in 2,965 patients. Patients undergoing TEVAR without arch debranching, TEVAR without inclusion diagnosis of cardiopulmonary bypass, TEVAR without inclusion criterion of median sternotomy were excluded. We then excluded all patients with diagnosis of emergent/emergent salvage or missing status for this diagnosis code. We also excluded those patients with diagnosis of acute aortic emergencies. Therefore, for a comparison dataset, we wanted to study elective total arch repair cases for diagnosis of aortic arch aneurysm, chronic dissection, and intramural hematoma. This resulted in 1,031 cases to be studied ( Fig. 1 ). Twenty of these cases were noted to have insufficient data and were therefore excluded from the analysis. This resulted in 1,011 cases performed at 274 centers across the nation, of which 884 patients underwent TAR with circulatory arrest (TAR group), and 127 patients had hybrid arch cases defined by diagnosis codes for aortic arch replacement and TEVAR (Hybrid Arch group). Technical aspects of surgical reconstruction for TAR and hybrid arch procedures are shown in Fig. 2 .

### Endpoints

The STS Adult Cardiac Surgery Database was analyzed for operative mortality, stroke, transient ischemic attack, renal failure, reoperation for bleeding/tamponade, paraplegia, composite STS major morbidity index, and composite mortality or stroke. Operative mortality was defined as operative death, discharge death, death before discharge, and death within 30 days after surgery. Composite STS major morbidity was defined as any of the following postoperative complication: deep sternal wound infection, renal failure, prolonged mechanical ventilation, reoperation for bleeding, and stroke.

### Statistical Analysis

Adjustment to several variables between the two groups was first attempted using propensity matching, but this left imbalance between the two groups. Therefore, stepwise selection was performed to narrow down the full risk set with 0.05 entry and 0.05 stay. Variables for risk adjustment included atrial fibrillation, body surface area, congestive heart failure class IV and nonclass IV, chronic lung disease, last creatinine, diabetes requiring insulin, ejection fraction, intra-aortic balloon pump, cardiogenic shock, age centered at 50 years, dialysis, female body surface area, hypertension, immunosuppression, peripheral vascular disease, myocardial infarction in last 21 days, reoperation status, age reoperation interaction, left main disease, active infection, and age centered at 75 years.

As no patient-level data were accessed by the investigators, institutional review board approval was waived.

## Results

### Cohort and Patient Selection

Overall, 1,011 cases performed at 274 centers across the United States were included in the analysis. The majority of cases ( *n* = 658/1,031) were performed for aneurysm indications. Dissection, intramural hematoma, or penetrating aortic ulcer were the second most common indication ( *n* = 373).

### Patient Demographics and Preoperative Parameters

Demographics and preoperative parameters are shown in Table 1 . Overall, patients undergoing hybrid arch procedures were older (mean age: 62.7 ± 13.3 vs 66.7 ± 11.9 years; *p* = 0.001), had higher rates of hypertension (87.4 vs. 94.9%; *p* = 0.02), peripheral vascular disease (34.1 vs. 49.6%; *p* = 0.0006), and renal failure requiring dialysis (1.7 vs. 4.7%; *p* = 0.03). Rates of congestive heart failure diagnosis (17.1 vs. 9.5%; *p* = 0.03) and aortic stenosis (10.5 vs. 3.9%; *p* = 0.02) were higher in patients undergoing TAR. Importantly, rates of history of cerebrovascular disease and stroke were similar between the two groups ( Table 1 ).

**Table 1:** Baseline characteristics

Variables	Overall ( *n* = 1,011)		TAR ( *n* = 884)		Hybrid ( *n* = 127)		*p* -Value
*Demographics* :							
Age	63.2 ± 13.2		62.7 ± 13.3		66.7 ± 11.8		**0.001**
Gender (female)	420	41.5%	366 (41.4)	41.4%	54	42.55%	0.811
*Race* :							
Native American	3	0.3%	2	0.2%	1	0.8%	
Asian	39	3.95	30	30%	9	7.1%	
Hispanic	41	4.1%	36	36%	5	3.9%	**0.016**
Black	117	11.6%	93	10.5%	24	18.9%	
Caucasian	764	75.6%	678	76.7%	86	67.7%	
Other	24	2.4%	23	23%	1	0.8%	
BSA (m ^2^ )	2.0		2.0	2.0	2.0		0.627
DM	136	13.5%	115	13.0%	21	16.5%	0.279
*DM control* :							
Insulin	21	15.4%	17	14.8%	4	19.1%	
Oral	62	45.6%	52	45.2%	10	47.6%	0.842
Diet	21	15.4%	19	16.5%	2	9.5%	
None	23	16.9%	19	16.5%	4	19.1%	
Hypertension	893	88.3%	773	87.4%	120	94.5%	**0.022**
Last creatinine	1.1		1.1		1.2		0.877
Dialysis	21	2.1%	15	1.7%	6	4.7%	**0.026**
*Chronic lung disease* :							
Mild	125	12.4%	113	12.8%	12	9.5%	0.782
Moderate	56	5.5%	45	5.1%	11	8.7%	
Severe	39	3.9%	34	3.9%	5	3.9%	
Immunosuppression	43	4.3%	35	4.0%	8	6.3%	0.213
PVD	364	36.0%	301	34.1%	63	49.6%	**<0.001**
CVD	210	20.8%	181	20.5%	29	22.8%	0.523
Endocarditis	10	1.0%	10	1.1%	0	0.0%	0.228
Smoking	114	11.3%	100	11.3%	14	11.0%	0.906
*Cardiac status* :							
Myocardial infarction	82	8.1%	69	7.8%	13	10.2%	0.303
CHF	163	16.1%	151	17.1%	12	9.5%	**0.027**
*NYHA class* :							
I	38	23.3%	34	22.5%	4	33.3%	
II	76	46.6%	73	48.3%	3	25.0%	0.424
III	36	22.1%	32	21.2%	4	33.3%	
IV	8	4.9%	7	4.6%	1	8.3%	
Unstable angina	15	1.5%	15	1.7%	0	0.0%	0.139
Cardiogenic shock	0	0%	0	0%	0	0%	N/A
Resuscitation	0	0%	0	0%	0	0%	N/A
Arterial fibrillation	151	14.9%	129	14.6%	22	17.3%	0.420
*Hemodynamics* :							
*Ejection fraction*							
≥ 45	859	93.0%	753	92.7%	106	94.6%	0.484
< 45	64	6.9%	58	7.1%	6	5.4%	
*Ejection fraction*							
≥ 60	515	55.7%	452	55.75%	63	56.3%	0.918
< 60	408	44.2%	359	44.2%	49	43.85%	
Left main disease (≥50%)	21	2.1%	19	2.2%	2	1.6%	0.960
Aortic stenosis	98	9.7%	93	10.5%	5	3.9%	**0.020**
Mitral stenosis	8	0.8%	7	0.8%	1	0.8%	0.994
Tricuspid insufficiency (moderate to severe)	79	7.8%	73	8.3%	6	4.7%	0.166
Mitral insufficiency (moderate to severe)	82	8.1%	74	8.4%	8	6.3%	0.424
*Mechanical cardiac assist device* :							
IABP	1	0.1%	1	0.1%	0	0%	0.705

Abbreviations: BSA, body surface area; CHF, congestive heart failure; CVD, cerebrovascular disease; DM, diabetes mellitus; IABP, intra-aortic balloon pump; NYHA, New York Heart Association; PVD, peripheral vascular disease; TAR, total arch replacement.

Note: Data presented as mean ± standard deviation or *n* (%).

The most common disease diagnosis was aneurysm in both cohorts (90.6%; Table 2 ), followed by diagnosis of dissection. Concomitant cardiac procedures were performed at higher rate in TAR group (60 vs. 34.6%; *p* < 0.0001), especially concomitant valve procedures ( Table 3 ). Concomitant descending thoracic procedures were performed at significantly higher rate in the Hybrid Arch group (18 vs. 59.1%; *p* < 0.0001).

**Table 2:** Elective outcomes

Variables	Overall ( *n* = 1,011)		TAR ( *n* = 884)		Hybrid ( *n* = 127)		*p* -Value
30-day/in-hospital mortality	75	7.4%	59	6.7%	16	12.6%	0 **.022**
STS major morbidity	356	35.2%	302	34.2%	54	42.5%	0.067
Deep sternal wound injection	6	0.6%	4	0.5%	2	1.6%	0.122
Stroke	80	7.9%	61	6.9%	19	15.0%	**0.002**
Permanent paralysis	25	2.5%	16	1.8%	9	7.1%	**0.002**
Prolonged ventilation	318	31.5%	269	30.4%	49	38.6%	0.067
New renal failure	52	5.1%	41	4.6%	11	8.7%	**0.045**
Cardiac reoperation (bleeding, valve, graft, other cardiac)	72	7.1%	60	66.8%	12	9.5%	0.281
*Composite outcomes* :							
Mortality and stroke	131	13.0%	101	11.4%	30	23.6%	**<0.001**
Mortality, stroke and paralysis	134	13.3%	102	11.5%	32	25.2%	**<0.001**
Mortality, stroke, paralysis and renal failure (dialysis)	149	14.7%	115	13.0%	34	26.8%	**<0.001**

Abbreviations: STS, Society of Thoracic Surgeons; TAR, total arch replacement.

Note: Data are presented as *n* (%).

**Table 3:** Disease, diagnosis, and concomitant procedures

Variables	Overall ( *n* = 1,011)		TAR ( *n* = 884)		Hybrid ( *n* = 127)		*p* -Value
*Concomitant cardiac procedures* :							
CABG	132	13.1%	117	13.2%	15	11.8%	0.656
Aortic valve	437	43.2%	416	47.1%	21	16.5%	**<0.001**
Mitral valve	20	2.0%	18	2.0%	2	1.6%	0.727
Other cardiac procedures	121	12.0%	109	12.3%	12	9.4%	0.350
Valve surgery	449	44.4%	427	48.3%	22	17.3%	**<0.001**
Tricuspid valve	6	0.6%	5	0.6%	1	0.8%	0.761
Concomitant aortic	878	86.8%	772	87.3%	106	83.5%	0.228
Root	246	24.3%	228	25.85	18	14.2%	**0.005**
Ascending aorta	793	78.4%	708	80.1%	85	66.9%	**<0.001**
Descending—proximal	234	23.1%	159	18.0%	75	59.1%	**<0.001**
Descending—mid	72	7.1%	35	4.0%	37	29.1%	**<0.001**
Descending—distal	61	6.0%	33	3.7%	28	22.0%	**<0.001**
*Incidence of cardiovascular (CV) interventions* :							
First CV surgery	585	57.9%	513	58.0%	72	56.7%	0.872
First CV reoperation	343	33.9%	297	33.6%	46	36.2%	
Second CV reoperation	59	5.8%	54	6.1%	5	3.9%	
*Previous arch procedure* :							
DHCA	794	78.5%	731	82.7%	63	49.6%	**<0.001**
Aneurysm	917	90.7%	802	90.7%	115	90.6%	0.950
Rupture	12	1.2%	11	1.2%	1	0.8%	0.657
Dissection	342	33.8%	296	33.5%	46	36.2%	0.554
Pseudoaneurysm	43	4.3%	35	4.0%	8	6.3%	0.222
Penetrating ulcer	18	1.8%	11	1.2%	7	5.5%	**<0.001**
Intramural hematoma	17	1.7%	16	1.8%	1	0.8%	0.402

Abbreviations: AV, aortic valve; CABG, coronary artery bypass grafting; DHCA, deep hypothermic circulatory arrest; MV, mitral valve; TAR, total arch replacement; TV, tricuspid valve.

Note: Data are presented as *n* (%).

### Postoperative Outcomes

In-hospital or 30-day operative mortality was significantly lower in the TAR group (6.7 vs. 12.6%; *p* = 0.02; Table 2 ). Rates of stroke (6.9 vs. 15%; *p* = 0.002) and new onset renal failure (4.6 vs. 8.7%; *p* = 0.04) were also significantly lower in the TAR group. Overall, STS composite major morbidity was similar (34.1 vs. 42.5%; *p* = 0.07). Deep sternal wound infection (0.4 vs. 1.6%; *p* = 0.12) and reoperation (6.8 vs. 9.5%; *p* = 0.28) rates were similar in the two groups. Permanent paralysis rates were significantly lower with TAR (1.8 vs. 7.1%; *p* = 0.001). Therefore, overall composite mortality, stroke, and permanent paralysis rate was more than two-fold higher in the Hybrid Arch group (11.5 vs. 25.2%; *p* < 0.0001).

### Risk Adjusted Multivariable Models

Multivariable regression analysis for variables associated with postoperative mortality are shown in Table 4 . Disease diagnosis (aneurysm versus dissection) or age at reoperation were not associated with increased mortality, but age at index operation (odds ratio [OR] = 1.12; *p* = 0.007), female by body surface area (OR = 0.04; *p* < 0.0001), a history of unstable angina (OR = 8.4; *p* = 0.0002), and renal function (OR = 2; *p* = 0.002) were all associated with increased mortality risk. Importantly, hybrid arch procedure compared with TAR was associated with increased mortality (OR = 1.91; *p* = 0.04). Variables associated with postoperative stroke included left main disease (OR = 5.15; *p* = 0.0005) and age centered at 75 years (OR = 1.13; *p* = 0.006; Table 5 ). Hybrid arch procedures were also associated with increased risk of stroke (OR = 2.3; *p* = 0.004). Variables associated with combined risk of stroke or mortality outcome are also shown in Table 5 .

**Table 4:** Multivariable odds ratios for mortality

Variables	Odds ratio	95% CI	*p* -Value
Hybrid arch versus TAR	1.91	1.01–3.61	**0.046**
Dissection/IMH/PAU versus aneurysm	0.95	0.53–1.69	0.865
Age (centered at 75 years)	1.12	1.03–1.22	**0.007**
Age by reoperation	1.02	0.99–1.04	0.166
Creatinine	2.00	1.30–3.09	**0.002**
Ejection fraction	0.95	0.91–0.98	**0.003**
Female by BSA	0.04	0.01–0.20	**<0.001**
Left main disease	0.24	0.05–1.10	0.067
Unstable angina	8.40	2.69–26.20	**<0.001**

Abbreviations: BSA, body surface area; CI, confidence interval; IMH, intramural hematoma; PAU, penetrating atherosclerotic; TAR, total arch replacement.

Note: Data are presented as OR (95% CI).

**Table 5:** Multivariable odds ratios for stroke and compound stroke/mortality

Variables	Odds ratio	95% CI	*p* -Value
*Stroke* :			
Hybrid arch versus TAR	2.30	1.30–4.09	**0.005**
Dissection/IMH/PAU versus aneurysm	1.52	0.90–2.55	0.117
Age (centered at 75 years)	1.13	1.04–1.24	0.006
Age by reoperation	1.01	0.99–1.03	0.450
Creatinine	1.62	1.00–2.64	0.051
Ejection fraction	0.96	0.92–0.99	**0.011**
Female by BSA	0.12	0.01–1.12	0.062
Left main disease	5.15	2.04–13.01	**<0.001**
Unstable angina	1.81	0.49–6.63	0.371
*Compound stroke/mortality* :			
Hybrid arch versus TAR	2.31	1.48–3.59	**<0.001**
Dissection/IMH/PAU versus aneurysm	1.25	0.80–1.95	0.331
Age (centered at 75 years)	1.12	1.04–1.20	**0.002**
Age by reoperation	1.02	1.00–1.04	**0.025**
Creatinine	1.94	1.22–3.11	**0.005**
Ejection fraction	0.95	0.92–0.99	**0.005**
Female by BSA	0.10	0.02–0.41	**0.001**
Left main disease	2.58	0.97–6.81	0.057
Unstable angina	2.84	1.04–7.75	**0.041**

Abbreviations: BSA, body surface area; CI, confidence interval; OR, odds ratio; PAU, penetrating atherosclerotic; TAR, total arch replacement.

Note: Data are presented as OR (95% CI).

Variables associated with reoperation for any indication included hybrid arch (OR = 1.57; *p* = 0.05), diagnosis of dissection versus aneurysm (OR = 1.69; *p* = 0.008), and age (OR = 1.07; *p* = 0.05; Table 6 ). STS composite major morbidity outcome was significantly associated with following variables: age (OR = 1.07; *p* = 0.03), renal function (OR = 1.79, *p* = 0.006), but not hybrid arch procedure (OR = 1.35; *p* = 0.19; Table 7 ).

**Table 6:** Multivariable odds ratios for reoperation

Variables	Odds ratio	95% CI	*p* -Value
Hybrid arch versus TAR	1.57	1.00–2.46	0.051
Dissection/IMH/PAU versus aneurysm	1.69	1.15–2.49	**0.008**
Age (centered at 75 years)	1.07	1.00–1.15	0.051
Age by reoperation	1.02	1.00–1.04	0.089
Creatinine	1.18	0.84–1.67	0.338
Ejection fraction	1.01	0.95–1.07	0.833
Female by BSA	0.41	0.14–1.19	0.100
Left main disease	0.19	0.03–1.30	0.091
Unstable angina	0.72	0.16–3.31	0.671

Abbreviations: BSA, body surface area; CI, confidence interval; IMH, intramural hematoma; OR, odds ratio; PAU, penetrating atherosclerotic; TAR, total arch replacement.

Note: Data are presented as OR (95% CI).

**Table 7:** Multivariable odds ratios for STS major morbidity

Variables	Odds ratio (95% CI)	*p* -Value
Hybrid arch versus TAR	1.35 (0.86–2.13)	0.195
Dissection/IMH/PAU versus aneurysm	1.18 (0.85–1.64)	0.310
Age (centered at 75 years)	1.07 (1.01–1.13)	**0.032**
Age by reoperation	1.02 (1.01–1.04)	**0.001**
Creatinine	1.79 (1.18–2.73)	**0.006**
Ejection fraction	0.99 (0.95–1.03)	0.554
Female by BSA	0.26 (0.10–0.69)	**0.007**
Left main disease	1.66 (0.64–4.26)	0.294
Unstable angina	0.99 (0.38–2.58)	0.982

Abbreviations: BSA, body surface area; CI, confidence interval; IMH, intramural hematoma; OR, odds ratio; PAU, penetrating atherosclerotic; STS, Society of Thoracic Surgeons; TAR, total arch replacement.

Note: Data are presented as OR (95% CI).

## Discussion

Unlike ascending aorta replacement or descending thoracic aorta replacement or TEVAR, which are associated with much lower risk of neurologic complications, arch procedures are associated with increased risk of stroke, transient ischemic attack, and temporary neurologic dysfunction. 6 7 Also, unlike ascending aortic pathologies, which are primarily associated with some degree of connective tissue disease, aortic arch aneurysms are more commonly associated with atherosclerotic disease, similar to descending aortic pathologies. Surgical intervention for atherosclerotic and connective tissue aneurysms in the ascending aorta and arch carry higher risk of complications than connective tissue disease, especially stroke/neurologic complications, most probably due to the calcific burden and the technical difficulties with aortic reconstruction associated with the former condition. 8 The national outcomes of arch procedures involving median sternotomy and cardiopulmonary bypass as documented by the STS database validate this finding, the overall mortality and stroke rates in the entire cohort were higher than that reported in the literature for DTA, TEVAR, or ascending aorta replacement procedures. Secondary to these high morbidities with DTA, hybrid arch replacement has been developed and practiced with increasing frequency.

TAR has been the gold-standard operation for management of aneurysm, dissection, and other arch pathologies requiring surgical intervention. Several groups have reported excellent postoperative outcomes with this operation, especially in the elective setting. 2 6 9 In high-risk patients, such those with advanced age and/or comorbid burden, hybrid arch procedures have been advocated as a safer alternative. 10 11 12 But most of these studies are single center experiences, presented as a retrospective review analysis.

In a recent study reviewing our institutional experience with hybrid arch versus TAR procedures, equivalent outcomes were noted between the two modalities, but decreased mortality was seen with hybrid arch procedures in older patients and those with a higher comorbid burden. 13

In a study by Iba et al, 10 the authors performed a propensity score-matching analysis of TAR ( *n* = 35) versus hybrid aortic arch ( *n* = 35) procedures and showed similar 30-day mortality. Moreover, they showed lower reintervention rates with TAR (1 vs. 20%); but a higher length of stay. 10 One criticism of that study was that the majority of patients undergoing hybrid arch procedures did not undergo any open debranching (36 out of 50). In another propensity score-matching, operative mortality was similar between the two groups, but at 24-month follow-up, hybrid arch procedures were associated with higher reintervention rates (1 vs. 21%). 12 This study suggested that hybrid arch procedures should be considered primarily in high-risk patients, where prolonged circulatory arrest periods may not be well tolerated.

A meta-analysis comparing TAR with hybrid arch procedures showed that operative mortality was similar (OR = 0.67), with a nonsignificant trend to higher neurologic complications with the hybrid arch group (OR = 1.93; 95% confidence interval [CI]: 0.86–4.37, *p* = 0.1). 2

A meta-analysis of hybrid arch procedures showed a 30-day mortality of 11.9%, stroke rate of 7.6%, and spinal cord injury rate of 3.6%. 14 A similar meta-analysis of TAR involving 21 studies reported a pooled mortality rate of 5.3%, stroke rate of 3.4%, and spinal cord injury rate of 0.6%. 6 Taken together, these studies suggested that hybrid arch procedures are associated with higher rates of stroke and spinal cord ischemia complications, although this patient population may comprise a higher risk cohort compared with patients undergoing TAR.

There are several aspects of the hybrid procedure itself that may contribute to these results. Advance endovascular skills are needed to accurately position and deploy the endograft which may result in increased circulatory arrest times. Further, positioning requires traversing the aortic arch with a large device which may contribute to observed neurological event rate. Finally, by performing a more extensive aortic replacement but adding a portion of the DTA to the operation the risk of paraplegia and spinal cord injury is increased.

The results of the current national investigation support the findings in the literature. Even after risk adjustment, we found that hybrid arch procedures were associated with a significantly higher risk of mortality and neurologic complications. However, other single institutional studies showed equivalent mortality outcomes, although several of these studies included patients who did not undergo arch vessel debranching into the hybrid arch cohort. Consequently, they may have included patients with zone-II proximal endograft landing, which can be accomplished safely with a carotid-subclavian artery bypass. In contrast, we only selected patients who electively underwent a median sternotomy with cardiopulmonary bypass and arch pathology diagnosis with a resultant TEVAR. Although this excludes patients who underwent type-I arch hybrid procedures performed without cardiopulmonary bypass and those undergoing staged hybrid arch procedures, we wanted to minimize heterogeneity in our patient populations. For example, a patient who underwent a carotid–to-subclavian bypass and subsequently underwent a TEVAR would have been captured in these data had we not excluded this population. Inclusion of these patients could potentially misrepresent the results and alter the study's aims.

While there is need for improvement in the data collection for these unique aortic arch cases, future versions of the STS database will likely include detailed information to facilitate meticulous investigations of arch procedure outcomes.

The results of our investigation highlight several aspects of optimal surgical management of aortic arch pathologies, especially since endovascular technology is being rapidly advanced to provide platforms for single and dual branch endografts for zone-0 landing. This is enhanced by the adoption of hybrid grafts (proximal Dacron graft with arch vessel branches and distal endograft) that enable simultaneous TAR with TEVAR of the DTA. “Empiric” stenting of the DTA for improved long-term remodeling of the DTA has been advocated for management of acute DeBakey Type-I aortic dissection. 15 16 17 Several single-institution retrospective studies, including ours, have shown technically tailored variations of this concept where stenting the DTA at the time of the arch procedure improves thoracic aortic remodeling. 17 18 19 20 21 Although these studies report excellent outcomes, hybrid arch procedures have not been directly compared with TAR in a prospective, randomized investigation. This multicenter investigation (274 centers in the United States) suggests that given the superiority of TAR over hybrid arch procedures, we need to exercise caution before rapid adoption and wide implementation of endovascular technologies for arch pathologies. Given the results of this study and the accumulating literature on hybrid arch procedures, we believe it is time for a prospective, multicenter, randomized trial comparing the two procedures to better understand optimal surgical management of arch pathologies. Currently, there are well-delineated algorithms for management of ascending and descending aortic pathologies, but aortic arch management in the era of TEVAR needs to be redefined through careful, unbiased investigation.

### Limitations

There are several limitations in analyzing patients undergoing TEVAR procedures. This group represents a very heterogeneous population that likely has had endografts placed for variable reasons and with variable amount of DTA coverage. Further, this group has more extensive coverage of the DTA as compared with the TAR group. Thus, drawing a strong conclusion from this group may be difficult.

This study utilized a national database that is still in development for specific hybrid arch treatment modalities and, as such, type-1 hybrid arch procedures were excluded. It is expected that the newer versions of the STS Adult Cardiac Surgery Database will allow more granular information on this exciting new procedure. The current study only evaluated early outcomes. It is plausible that longer term follow-up will lead to a better understanding of the aortic remodeling that is achieved and possible aortic reintervention rates with each procedure type.

## Conclusion

As endovascular technology continues to improve and push the limits of the treatment of aortic arch, the field of cardiac surgery will also need to evolve to make endovascular training an integral part of teaching modules/programs for current practicing cardiac surgeons and fellows/residents in training. This will facilitate our field's widely and safely adopting and investigating the role of endovascular and hybrid techniques in aortic arch surgery. This will also prepare us to independently treat aortic arch pathologies.
